# Autism Spectrum Disorder–Associated Genes Enrich in Discrete Cortical, Limbic and Cerebellar Brain Regions

**DOI:** 10.1111/ejn.70638

**Published:** 2026-07-24

**Authors:** G. Lorenzo Odierna, Vicki Bitsika, Christopher F. Sharpley

**Affiliations:** ^1^ Brain‐Behavior Research Group University of New England Armidale New South Wales Australia

**Keywords:** autism spectrum disorder, biomarkers, brain regions, early diagnosis, gene expression, neurodevelopmental disorder, synaptic dysfunction

## Abstract

Autism spectrum disorder (ASD) is a highly heritable neurodevelopmental condition with a well‐characterised genetic architecture, yet how ASD‐associated genes map onto discrete brain regions remains poorly understood. This study applied a recently validated analysis pipeline (ATLANTE) to discover regions of the human brain enriched for high expression of 234 high‐confidence ASD‐associated genes. Eleven discrete brain regions were identified, spanning cortical, limbic and cerebellar systems including the anterior orbitofrontal gyrus, cerebellar cortex, flocculonodular lobe, vermis, posterior cingulate cortex, temporo‐occipital transitional zone, hippocampal subfields CA1 and CA3, postcentral gyrus, occipital cortex and perirhinal gyrus. Notably, the anterior orbitofrontal gyrus exhibited significant enrichment for syndromic ASD genes, suggesting a core role in the neuropsychiatric features of syndromic presentations. Network and community clustering analyses revealed three major gene communities corresponding to distinct biological processes: synaptic dysfunction in limbic regions, histone modification in cerebellar regions and axonal ion channel regulation in cortical regions. Nodal analysis identified eight high‐priority genes with broad relevance across multiple brain regions, including *NR3C2*, *GABRB2* and *NBEA*. These findings provide a refined neuroanatomical framework for ASD pathophysiology, identify understudied brain regional targets and imply that ASD‐associated genes exert primary effects in specific brain regions.

## Graphical abstract

This study identified 11 brain regions enriched for ASD‐associated genes, spanning limbic, cerebellar and cortical systems. Network analysis revealed region‐specific dysfunction: synaptic dysregulation in limbic areas, histone modification in the cerebellum and axonal ion channel disruption in the cortex. Several genes, including NR3C2, were identified as high‐priority targets for further study.

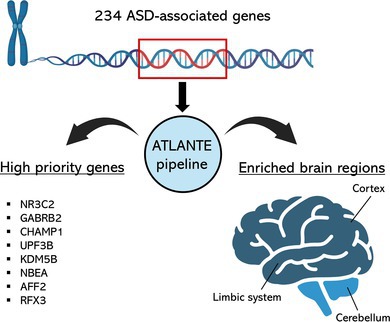

## 1 Introduction

Autism spectrum disorder (ASD) is a neurodevelopmental condition characterised by persistent impairments in social communication, restricted interests and repetitive behaviours (Hirota and King 2023). The clinical severity of ASD is notably heterogeneous, ranging from individuals requiring substantial support to those with minimal support needs. ASD is often accompanied by co‐occurring conditions including intellectual disability (ID), attention‐deficit/hyperactivity disorder, epilepsy and anxiety disorders (Masi et al. 2017). This heterogeneity extends beyond clinical symptoms to encompass diverse neurobiological profiles (Liu et al. 2025), complicating efforts to develop unified models of ASD neurobiology.

A major advance in understanding ASD has come from genetic research, which has established that ASD is highly heritable, with most estimates producing values around 80% (Bai et al. 2019; Sandin et al. 2017) (heritability here is defined as the variance in phenotypic expression explained by genetics). Over the past two decades, genome‐wide association studies (GWAS), whole‐exome sequencing and large‐scale consortia such as the Simons Foundation Autism Research Initiative (SFARI) have identified hundreds of genes confidently associated with ASD (Grove et al. 2019; Abrahams et al. 2013; Yu et al. 2013). These genes span diverse cell and molecular functions including synaptic transmission, chromatin remodelling, transcriptional regulation and neuronal development (Rylaarsdam and Guemez‐Gamboa 2019). Despite this wealth of genetic information, a critical gap remains, which is to understand where in the brain these genes exert their primary effects and how genetic heterogeneity maps onto the neurobiological and clinical heterogeneity observed in ASD.

Many brain regions have been implicated in ASD through structural and functional neuroimaging studies, including the cortex (Ha et al. 2015), thalamus (Ayub et al. 2021), limbic structures (Li et al. 2023), cerebellum (Mapelli et al. 2022), basal ganglia (Prat et al. 2016) and brainstem (Seif et al. 2021). However, it remains unclear which of these regions represent primary loci of dysfunction, where ASD‐associated genes are directly influencing development and function, versus secondary effects arising from disrupted connectivity or compensatory mechanisms. Gene ontology (GO) analyses of ASD‐associated genes have consistently identified enrichment for many cell and molecular processes (Rahnama et al. 2021), but these analyses often treat the brain as regionally monotypic. Although this often stems from challenges associated with sampling from multiple loci, it potentially de‐emphasises the importance of region‐specific signatures that ultimately must be addressed to gain meaningful mechanistic insights into the neurobiology of ASD.

One promising approach to bridge genetic data with neuroanatomical specificity is to identify brain regions enriched for high expression of ASD‐associated genes. If genes linked to ASD share high expression in discrete brain regions, this may indicate that those regions are particularly vulnerable to genetic perturbations and thus represent primary sites of dysfunction. Such a gene‐informed approach could help operationally prioritise brain regions for detailed investigation, guide the development of region‐specific biomarkers for early diagnosis and inform our understanding of how diverse genetic aetiologies converge onto common neural circuits.

To address this gap, the current study aimed to apply ATLANTE (Analysis Tool for Local Association of Neuronal Transcript Expression), a recently developed and validated analysis pipeline (Odierna, Sharpley, et al. 2025), to identify brain regions enriched for high expression of high‐confidence ASD‐associated genes. ATLANTE operates by comparing the observed frequency of highly expressed genes from a user‐defined list across 193 discrete brain regions against reference distributions generated via Monte Carlo simulation. The pipeline has been previously validated using positive control gene lists and successfully identified novel brain regional targets based on genes associated with major depression (Odierna, Sharpley, et al. 2025).

In this study, ATLANTE was applied to 234 high‐confidence ASD‐associated genes curated from the SFARI Gene database, encompassing both syndromic and non‐syndromic forms of ASD. The aims of this study were to identify discrete brain regions with significant enrichment of ASD‐associated genes, explore relationships between identified brain regions and characterise region‐specific biological processes through GO analysis.

## 2 Material and Methods

### 2.1 ASD‐Associated Gene List Selection

Genes of interest were sourced based on criteria determined by the human gene module of the SFARI Gene database (https://gene.sfari.org/). This database is a continuously updated platform that consolidates results from peer‐reviewed research reports on genetic variants relevant to ASD. Genes are organised based on confidence (scale of 1–3) and functional category (‘rare’, ‘syndromic’, ‘association’ or ‘functional’). For this study, only genes listed as being ‘Category 1’ (high confidence) were selected. As defined by SFARI, Category 1 genes must be implicated in ASD by at least three de novo mutations and/or have crossed genome‐wide significance by meeting a false discovery threshold of less than 0.1. Both syndromic and non‐syndromic genes were used in this study. Syndromic genes are defined by SFARI as being consistently linked to additional characteristics not required for an ASD diagnosis but are strongly associated with ASD (such as fragile X syndrome or Rett syndrome). The total number Category 1 genes was 234. Of these, the number of non‐syndromic genes was 118 and the number of syndromic genes was 116 (full gene list shown in Table S1).

### 2.2 Analysis Using the ATLANTE Pipeline

The process by which ATLANTE operates has been previously described and validated (Odierna, Sharpley, et al. 2025). The source code for ATLANTE is publicly accessible online at https://github.com/LorenzoOdierna/Analysis‐Tool‐for‐Local‐Association‐of‐Neuronal‐Transcript‐Expression‐ATLANTE‐. In brief, ATLANTE is an analysis pipeline coded in python that identifies brain regions with statistically significant over‐representation of relatively high transcript expression for any given user‐generated list of genes. Here, a region was defined as having relatively high transcript expression by demonstrating expression (per gene) that was greater than 1.96 standard deviations of the mean across all brain regions. ATLANTE uses RNA transcript counts from 193 unique brain regions (data courtesy of Human Protein Atlas version 24.0, www.proteinatlas.org (Uhlén et al. 2015)). The dataset is derived from human brain region tissue samples isolated via microdissection and analysed using an MGI DNBSEQ‐17. Post‐mortem samples from each brain region represent averages generated across diverse pools of mixed male and female samples. The number of biological replicates per brain region ranged from 1 to 13 (per‐region mean = 5.3, standard deviation = 2.6), and the age of donors ranged from 18 to 94 (per‐region mean = 75.9, standard deviation = 6.4). In this analysis, raw transcriptional data are not directly tested for statistical significance but instead are used to generate representative normalised counts per brain region for further analysis.

In this study, the user‐generated list was comprised of ASD‐associated genes (Table S1). The observed distribution of ASD‐associated genes across all 193 brain regions was calculated and compared to a reference distribution (previously referred to as ‘reference counts’ (Odierna, Sharpley, et al. 2025)) in order to determine statistical significance. The reference distribution was generated via Monte Carlo simulation with 1,000,000 iterations. In each iteration, 234 random genes were selected from the 20,162 genes available in the Human Proteome Atlas dataset (roughly representative of the entire human genome), and expression frequency was calculated across all 193 brain regions. This process generated a reference distribution representing the expected regional (high) expression pattern for any random set of 234 genes. The frequency distribution of the ASD‐associated gene list was compared to the reference distribution using a Bonferroni‐corrected threshold of *p* < 0.05, with each separate brain region treated as an independent comparison. Fold‐enrichment values were determined for significantly enriched regions by calculating the ratio of the observed to expected frequencies (observed ASD gene count/simulated mean gene count).

To determine whether specific brain regions exhibited statistically anomalous proportions of syndromic versus non‐syndromic ASD genes, a hypergeometric distribution test was applied to each of the 11 brain regions identified in the ATLANTE analysis. For each brain region, a two‐tailed *p*‐value was calculated and corrected by applying a Benjamini–Hochberg false discovery rate (FDR) correction. Regions with FDR‐corrected *p* < 0.05 were considered statistically significant.

### 2.3 Generation of the ASD Gene–Brain Region Network Graph and Community Clustering

To examine the relationship between ASD‐associated genes and ATLANTE‐identified brain regions, a network graph was constructed such that nodes represented individual genes and edges represented shared brain regions where both genes in a pair exhibited relatively high transcript expression. This analysis included only the 178 genes and 11 brain regions identified through ATLANTE using the curated ASD‐associated gene list. A 178 × 11 gene–brain region matrix was generated where each entry indicated whether a gene was ‘present’ within a brain region (defined as relatively high transcript expression, as per Section 2.2). Using this matrix, all 15,753 possible gene pairs were assessed for shared presence across the identified brain regions, assigning a value of 0 for no shared presence and 1 for shared presence. When gene pairs shared multiple brain regions, the number was summed (weighted edge).

Nodal strength was calculated by summing all weighted edges for each node, betweenness centrality was calculated by summing the fractions of all shortest path pairs that passed through each node, and closeness centrality was calculated as the average shortest path length from each node to other reachable nodes (Barrat et al. 2004; Freeman 1978).

### 2.4 Network Clustering and Community GO Analysis

To identify emergent features potentially indicating shared or independent molecular identities among the enriched brain regions, cluster analysis was performed on the gene region network graph. A Louvain clustering algorithm (Blondel et al. 2008) was applied to identify large‐scale, nonoverlapping communities without requiring prior assumptions about cluster structure. Although this approach is less sensitive to small, granular community features, it minimises bias and yields more conservative results.

Gene lists derived from Louvain clustering annotations were analysed for GO enrichment using ShinyGO (Ge et al. 2020) v0.84. Statistically significant biological process (BP), molecular function (MF) and cellular component (CC) terms were identified using an FDR threshold of 0.05.

### 2.5 Data Processing, Statistical Analysis and Visualisation

Data handling, processing, analysis and visualisation were conducted using Jupyter Notebooks (Kluyver et al. 2016). The following python packages were used in this study: pandas (McKinney 2010) v2.2.2, NumPy (Harris et al. 2020) v2.3.4, SciPy (Virtanen et al. 2020) v 1.13.1, NetworkX (Hagberg et al. 2008) v3.5, Matplotlib (Hunter 2007) v3.9.2 and Plotly (Sievert 2020) v6.3.1.

## 3 Results

### 3.1 Enrichment of ASD‐Associated Genes in 11 Distinct Brain Regions

**FIGURE 1:**
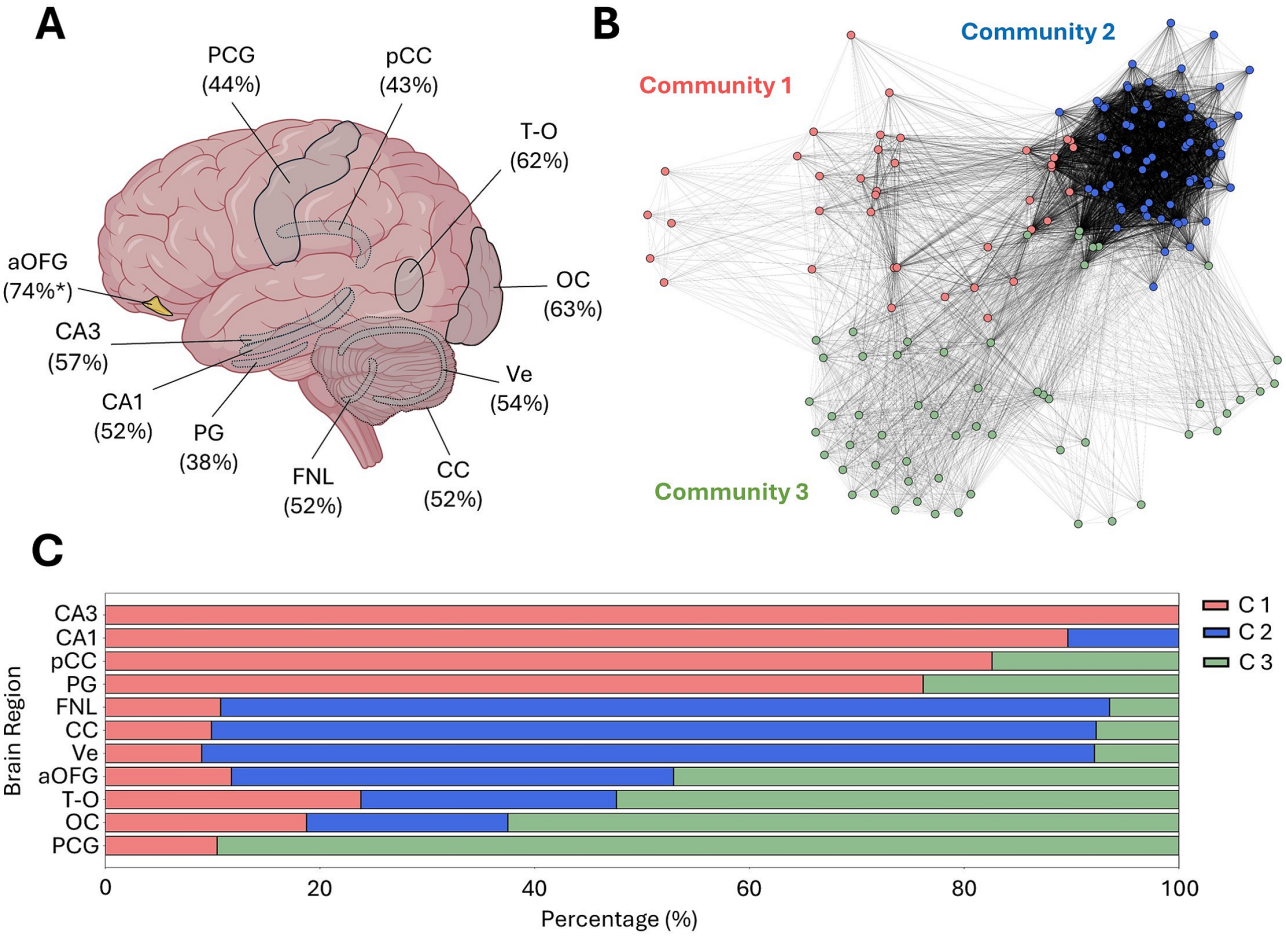
ASD genes share high expression in distinct regions of the brain. (A) Schematic illustration depicting brain regions that passed Bonferroni‐corrected threshold for statistical significance following application of the ATLANTE pipeline on a list of high‐confidence ASD‐associated genes. Numbers in parentheses are the percentage of syndromic (vs. non‐syndromic) ASD‐associated genes that were enriched in each brain region; yellow colouring and * signify that this value represents a statistically significant enrichment of syndromic genes, as determined by a hypergeometric test. (B) Network graph constructed with the goal of visualising ASD‐associated genes that enriched in the ATLANTE analysis as nodes and shared brain regions of relatively high expression as edges. Edge thickness (weight) determined by number of shared brain regions. Node colours represent the results of an unbiased Louvain clustering analysis that discovered three broad communities within the network. (C) Bar graph displaying the percentage of genes belonging to each community per brain region. C1 = Community 1, C2 = Community 2, C3 = Community 3. aOFG = anterior orbitofrontal gyrus, CA1 = cornu ammonis subfield 1 of the hippocampus, CA3 = cornu ammonis subfield 3 of the hippocampus, CC = cerebellar cortex, FNL = flocculonodular lobe, OC = occipital cortex, pCC = posterior cingulate cortex, PCG = postcentral gyrus, PG = perirhinal gyrus, T‐O = temporo‐occipital transitional zone, Ve = vermis.

Though the genetic architecture of ASD has been increasingly well mapped (Rylaarsdam and Guemez‐Gamboa 2019), interpreting this knowledge with the goal of generating holistic models of brain structure and function has been challenging. To address this gap, the present study applied the ATLANTE, which is a novel, validated analysis pipeline that can identify regions of the human brain enriched for relatively high expression of user‐defined lists of genes (see Section 2.2) (Odierna, Sharpley, et al. 2025). Here, ATLANTE was applied using a high‐confidence list of ASD‐associated genes, notably all Category 1 genes curated by the SFARI Gene database. The results of this analysis revealed that 11 discrete brain regions demonstrate a statistically significant enrichment of highly expressed ASD‐associated genes. The brain regions identified here include the anterior orbitofrontal gyrus (aOFG), cerebellar cortex (CC), flocculonodular lobe (FNL), vermis (Ve), posterior cingulate cortex (pCC), temporo‐occipital transitional zone (T‐O), cornu ammonis 3 subfield of the hippocampus (CA3), cornu ammonis 1 subfield of the hippocampus (CA1), postcentral gyrus (PCG), occipital cortex (OC) and perirhinal gyrus (PG; Table 1 and Figure 1A). These results indicate that expression of ASD‐associated genes is not randomly distributed across brain regions, but instead, they share relatively high expression in distinct regions of the brain.

**TABLE 1:** Brain regions significantly enriched for ASD‐associated genes.

Brain region	Abbreviation	*p*	Fold enrichment
Anterior orbitofrontal gyrus	aOFG	1.12 × 10^−10^	2.76
Cerebellar cortex	CC	7.11 × 10^−10^	1.74
Flocculonodular lobe	FNL	8.1 × 10^−10^	1.72
Vermis	Ve	3.08 × 10^−9^	1.71
Posterior cingulate cortex	pCC	5.34 × 10^−8^	2.83
Temporo‐occipital transitional zone	T‐O	9.59 × 10^−8^	2.91
Hippocampus, CA3	CA3	2.34 × 10^−6^	2.27
Hippocampus, CA1	CA1	5.93 × 10^−6^	2.16
Postcentral gyrus	PCG	8.02 × 10^−6^	1.78
Occipital cortex	OC	8.37 × 10^−5^	2.46
Perirhinal gyrus	PG	2.15 × 10^−4^	2.08

*Note:* Fold enrichment represents the ratio of observed ASD gene count to the expected count from Monte Carlo simulation (observed/expected). Values > 1 indicate enrichment above random expectation.

ASD‐associated genes can be classified based on whether they are linked to syndromic or non‐syndromic forms of the disorder. High‐confidence genes linked to both syndromic and non‐syndromic ASD were used in the ATLANTE analysis. To test whether any of the identified brain regions were specifically associated with syndromic or non‐syndromic ASD‐associated genes, a hypergeometric distribution test was applied. Each brain region was assessed based on the number of syndromic and non‐syndromic ASD‐associated genes that were strongly expressed. Out of all 11 identified brain regions, the aOFG displayed a statistically significant FDR‐corrected enrichment of syndromic ASD‐associated genes (*p* < 0.05; 74% syndromic vs. non‐syndromic; Figure 1A). Though the orbitofrontal cortex has already been implicated in ASD (Girgis et al. 2007), this finding suggests that the aOFG might be particularly important in syndromic manifestations of ASD.

### 3.2 Gene Region Network Clustering Reveals 3 Major Communities

To explore the relationship between the identified brain regions and the genes enriched in each of these, a graph theory approach was pursued. A network was constructed where individual genes were represented as nodes and shared brain regional enrichment between genes were represented as weighted edges (see Section 2.3 for additional details). The resultant graph demonstrated a complex topology, indicative of both shared and distinct gene signatures between brain regions. Louvain hierarchical clustering of the network (Blondel et al. 2008) supported this interpretation, with at least three major communities identified (Figure 1B).

To gain insight into how these communities mapped onto the brain regions identified by ATLANTE, each gene was annotated based on its community identity. The percentage of each community per brain region was calculated, revealing clear trends that appeared to associate individual brain regions together (Figure 1C). For example, although Community 1 genes were present in all brain regions, they exhibited an over‐representation in the CA3 (100% Community 1 genes), CA1 (89%), pCC (82%) and PG (76%). Conversely, Community 2 genes were consistently well represented in the FNL (83% Community 2 genes), Ve (83%) and CC (82%), whereas Community 3 genes were most strongly represented in the PCG (89% Community 3 genes), OC (62.5%), T‐O (52%) and aOFG (47%). These communities, which arose via an unbiased and unsupervised analysis of the shared ASD‐associated genetic overlap between brain regions, independently categorised the 11 brain regions into three high‐level systems: limbic (Community 1), cerebellar (Community 2) and cortical (Community 3). Though this finding may be possibly explained by common expression patterns at the tissue level due to shared cell lineages, it follows that these three systems likely have a unique, fundamental relevance to ASD.

### 3.3 Identification of High‐Priority Genes via Nodal Analysis

Networks are amenable to analyses that can interrogate the relational properties of individual nodes. Nodes that are highly interconnected, located centrally or serve as bridges between hubs hold special value in the network, and as such, identifying them can often reveal key insights. In the case of the ASD gene region network, highly and centrally interconnected nodes represent genes that are highly expressed across many of the identified brain regions and thus could be seen as having more widespread or generalisable relevance to ASD.

**FIGURE 2:**
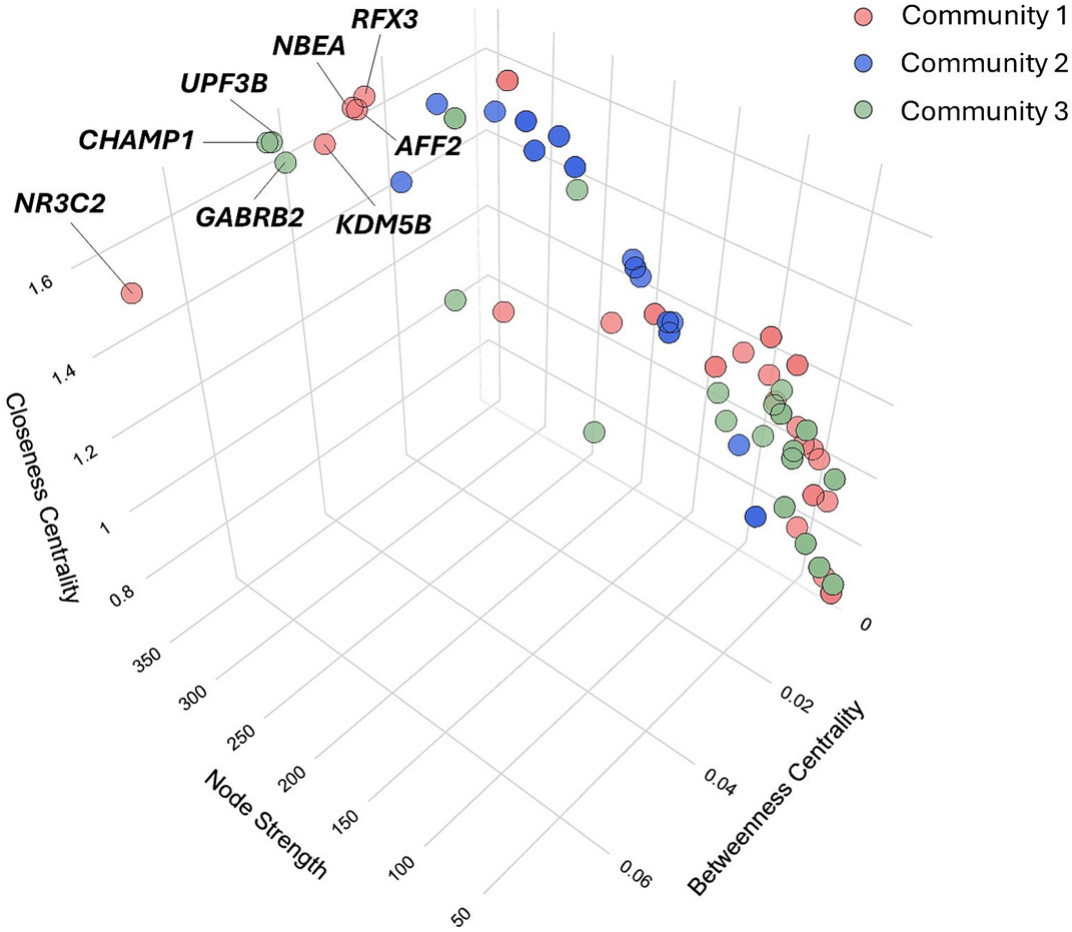
High‐scoring genes across several nodal network analysis measures. 3D scatterplot where each point represents a node (gene) in the brain region ASD network. Data are plotted based on calculated scores of node strength, betweenness centrality and closeness centrality. The top 5 genes across each measure are indicated within the graph. Nodes are coloured based on previously identified communities following Louvain clustering.

Node strength, closeness centrality and betweenness centrality were calculated for each node in the network (see Section 2.3). To visualise the results for each node (gene), a 3D scatterplot was generated with the three axes representing node strength, closeness centrality and betweenness centrality (Figure 2). The results indicated that nodes within the network displayed a broad range of properties but a select few exhibited high values for all three measures. Indeed, the top 5 genes for node strength, closeness centrality and betweenness centrality were populated by only 8 genes in total: *NR3C2*, *GABRB2*, *CHAMP1*, *UPF3B*, *KDM5B*, *NBEA*, *AFF2* and *RFX3* (Figure 2). Re‐examination of the positions of these nodes in the network confirmed that their high scores across all three metrics reflected each of them sharing many strong connections with nodes across all three of the previously identified Louvain communities. As such, the eight genes identified here may hold broad relevance to ASD by being highly expressed across many critical brain regions.

### 3.4 Community‐Specific Biological Processes, Molecular Functions and Cellular Components

To date, several hundreds of genes have been associated with ASD (Rylaarsdam and Guemez‐Gamboa 2019). Gene ontological analysis of these often yields results suggesting that dysregulation of synaptic plasticity, metabolism and cell cycle regulation underlie the expression of ASD symptoms (Rahnama et al. 2021; Kereszturi 2024). It stands to reason that each of these ontological signatures has discrete sources tracing back to specific cellular populations or tissue subregions because (1) gene expression across different cell types and brain regions is heterogeneous and (2) the gene lists used for ontological analysis are often determined by detecting polymorphisms or mutations at the genome level, which does not comment on expression patterns. There have been many increasingly sophisticated approaches to exploring the cell type–specific source of ontological signatures, such as by employing single‐cell RNA sequencing (Wang et al. 2018; Wamsley et al. 2024). Though these often successfully identify cell type–specific sources of the signatures, tissue subregional sources have yet to be explored in detail. To address this, the list of ASD‐associated genes highly expressed in the three Louvain communities identified in this study was subjected to GO analysis with the goal of identifying tissue subregion‐specific signatures.

**FIGURE 3:**
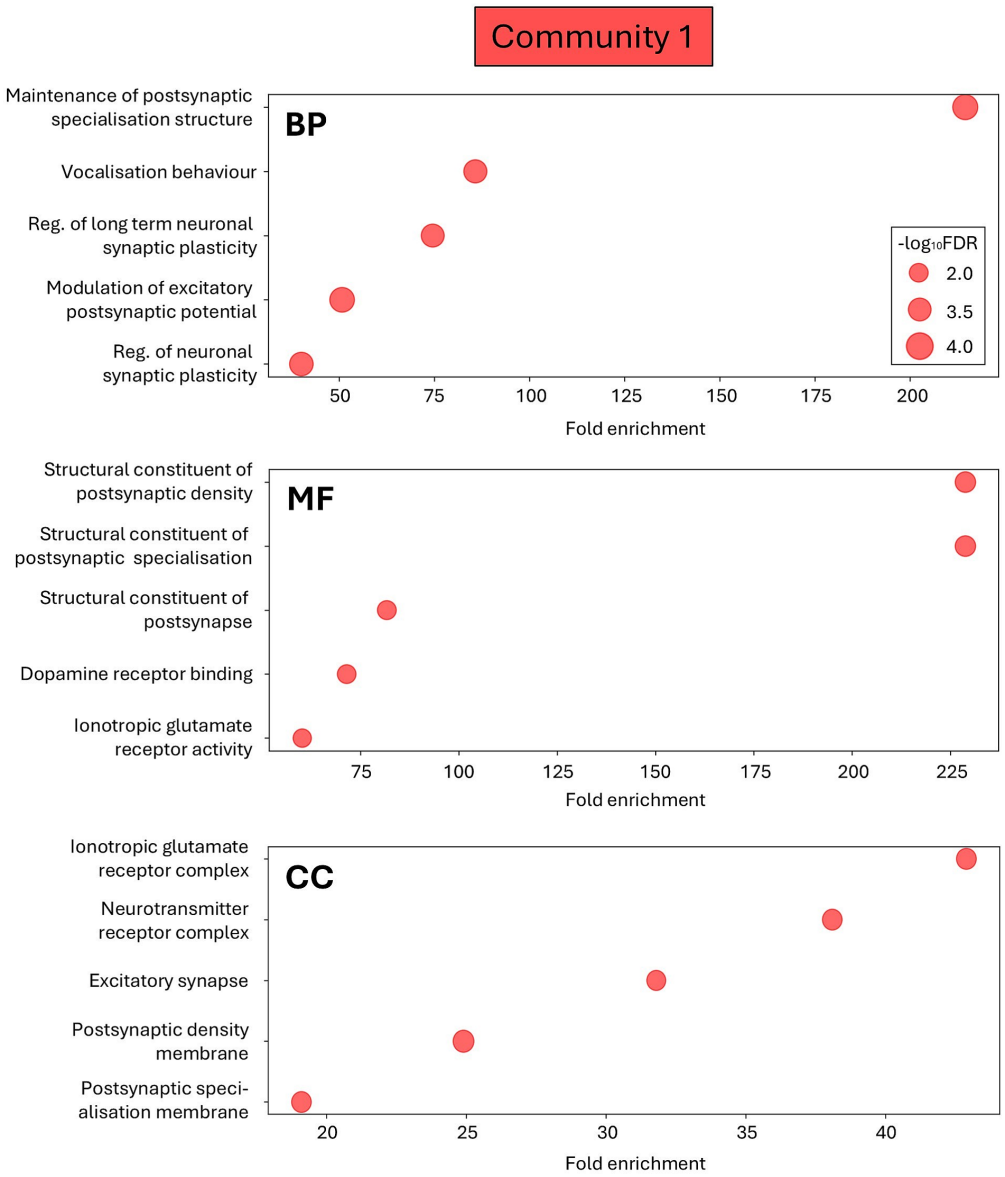
Community 1 gene ontology analysis. Gene ontology analysis was performed for the Community 1 gene list following Louvain clustering. The top 5 term results for biological process (BP), molecular function (MF) and cellular component (CC) are shown. Each term is plotted as a circle along the x‐axis, representing fold enrichment. The size of individual circles represents −log_10_FDR (key shows rough mapping of circle size to −log_10_FDR).

**FIGURE 4:**
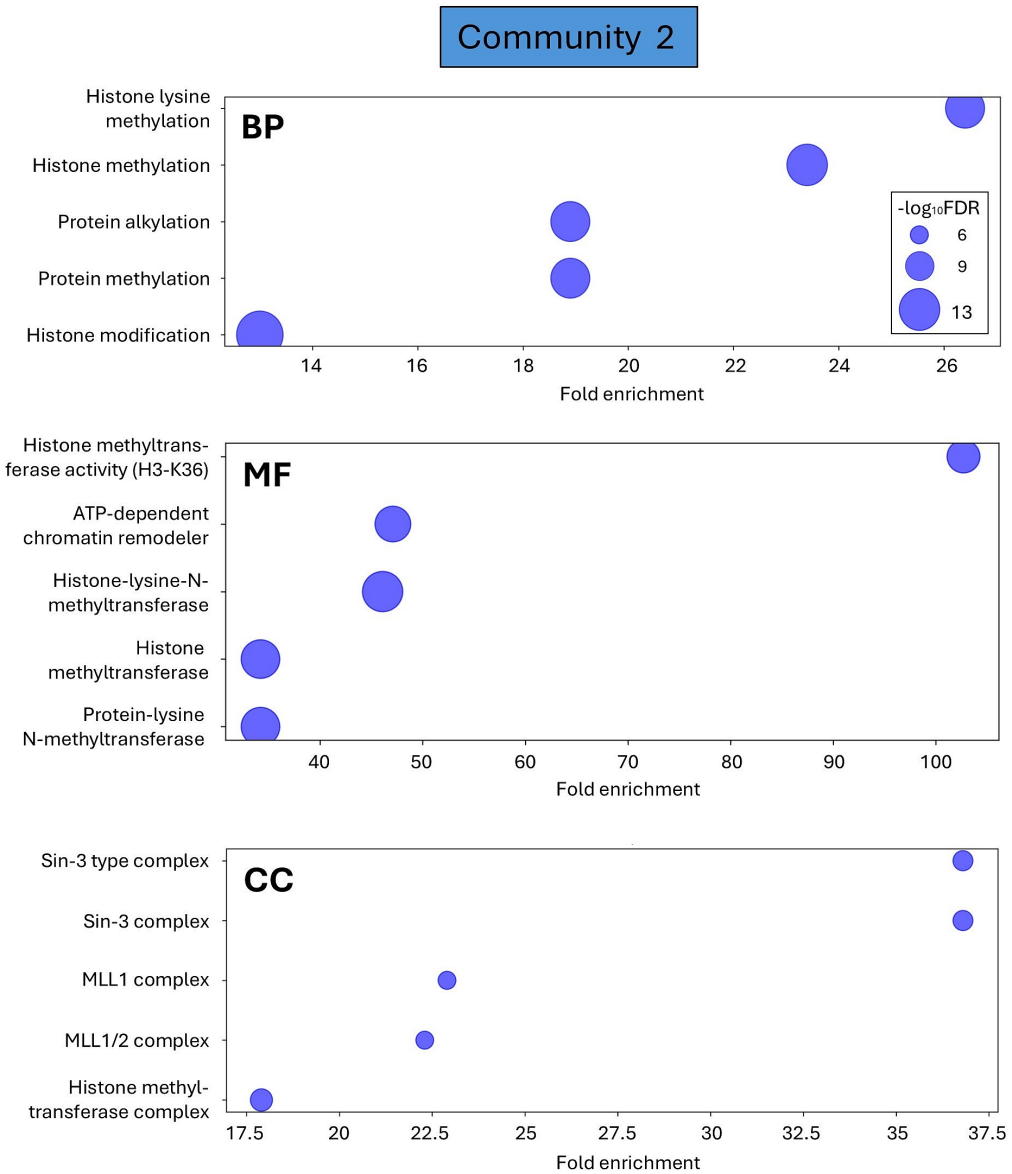
Community 2 gene ontology analysis. Gene ontology analysis was performed for the Community 2 gene list following Louvain clustering. The top 5 term results for biological process (BP), molecular function (MF) and cellular component (CC) are shown. Each term is plotted as a circle along the x‐axis, representing fold enrichment. The size of individual circles represents −log_10_FDR (key shows rough mapping of circle size to −log_10_FDR).

**FIGURE 5:**
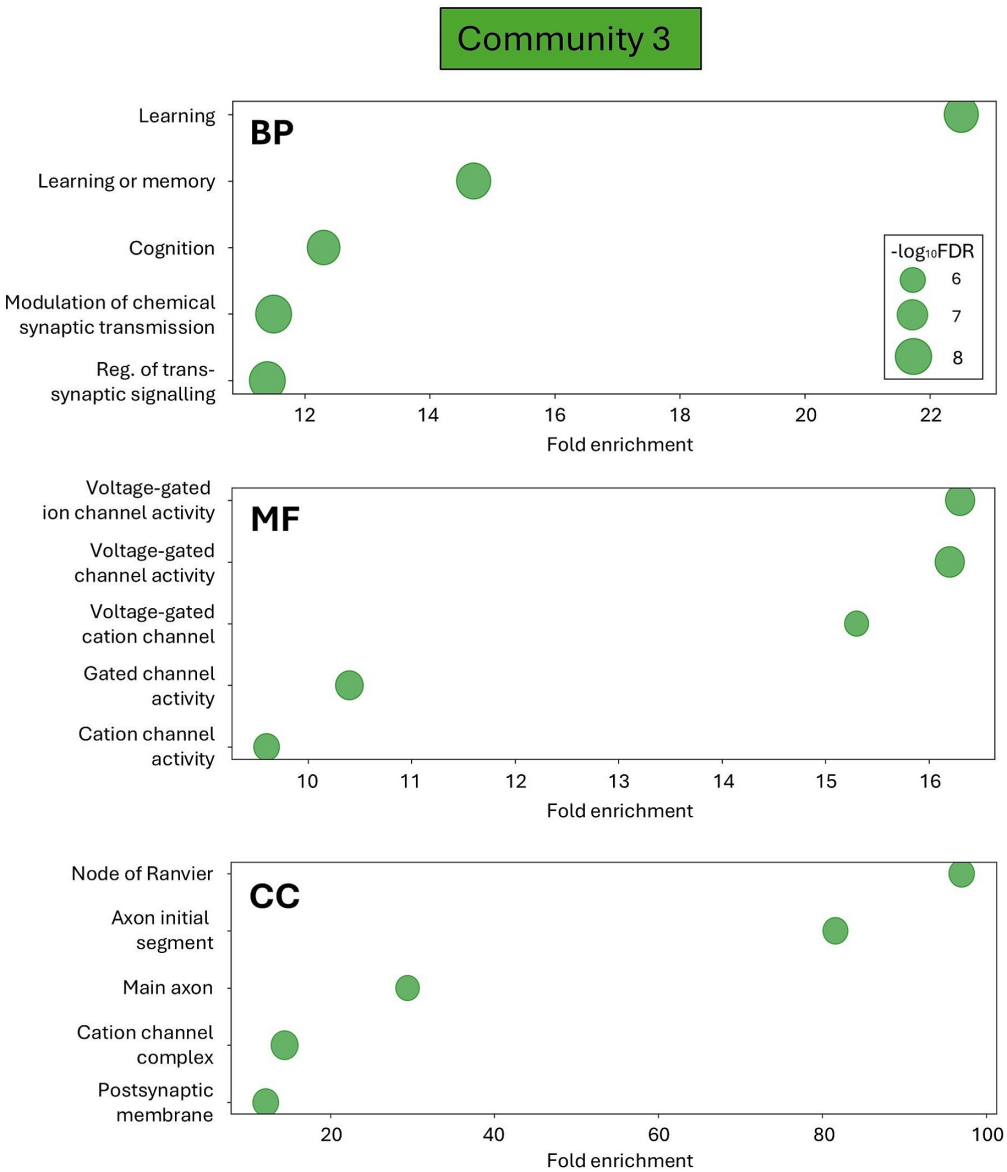
Community 3 gene ontology analysis. Gene ontology analysis was performed for the Community 3 gene list following Louvain clustering. The top 5 term results for biological process (BP), molecular function (MF) and cellular component (CC) are shown. Each term is plotted as a circle along the x‐axis, representing fold enrichment. The size of individual circles represents −log_10_FDR (key shows rough mapping of circle size to −log_10_FDR).

GO analysis of each community's gene list returned statistically significant FDR‐corrected terms. Interestingly, the terms associated with each community's gene list were discrete, indicating a bias in brain subregion‐specific dysfunction. Community 1, which was particularly associated with the limbic system (Figure 1C; brain regions: CA1, CA3, PG and pCC), was generally enriched for biological function terms associated with synaptic function and plasticity (Figure 3). Molecular function terms reflected this, as did cellular component terms, which all centred around structural regulation of synapses and postsynaptic membrane complexes (Figure 3). Notable terms to ASD symptoms included the biological process, ‘vocalisation behaviour’, and the molecular function, ‘dopamine receptor binding’. Community 2, which was associated with the cerebellum (Figure 1C; brain regions: CC, FNL and Ve), was instead enriched for biological process and molecular function terms for histone modification and protein post‐translational modification (alkylation and methylation, in particular; Figure 4). Cellular component terms for Community 2 largely reflected this and further indicated enrichment with the Sin3 histone deacetylase and HLL1 methyltransferase complexes (Figure 4). Community 3, which was associated with cortical regions (Figure 1C; brain regions: PCG, OC, T‐O and aOFG), was enriched for biological function terms relating to learning, memory, cognition and modulation of synaptic transmission (Figure 5). Molecular function terms enriched for Community 3 genes included voltage‐gated channel activity, specifically cation channels. Interestingly, cellular component terms were strongly indicative of axonal processes, with terms specifically relating to nodes of Ranvier and the axon initial segment (Figure 5). These findings demonstrate that there are significant brain regional differences in the types of processes influenced by ASD‐associated genes, suggesting that the fundamental nature of the dysfunction in these regions, and how they manifest at a cell and molecular level, is likely distinct.

## 4 Discussion

### 4.1 ASD‐Associated Genes Are Enriched in Discrete Brain Regions

Translation of the increasingly well‐understood genetic architecture of ASD into meaningful predictions about fundamental drivers of altered brain structure and function is a major goal in ASD research. This translation is an indispensable step towards discovering novel biomarkers for confident and early diagnosis, as well as generating therapeutic strategies that can positively influence the quality of life of autistic individuals. In this study, an analysis pipeline called ATLANTE was used to identify brain regions enriched for high expression of genes associated with ASD, with the goal of identifying core diagnostic biomarker targets and discrete loci with mechanistically causal significance to ASD. ATLANTE operates by comparing the frequency (count) of highly expressed genes per brain region, given a user‐generated gene list, against reference frequencies generated via Monte Carlo simulation. The pipeline has been previously validated and has successfully identified distinct brain regions based on genes associated with major depression (Odierna, Sharpley, et al. 2025). Here, application of ATLANTE using a list of high‐confidence ASD‐associated genes produced 11 discrete brain regions. These regions (in order of corrected *p*‐value) were the anterior orbitofrontal gyrus (aOFG), cerebellar cortex (CC), flocculonodular lobe (FNL), vermis (Ve), posterior cingulate cortex (pCC), temporo‐occipital transitional zone (T‐O), cornu ammonis subfield 3 of the hippocampus (CA3), cornu ammonis subfield 1 of the hippocampus (CA1), postcentral gyrus (PCG), occipital cortex (OC) and perirhinal gyrus (PG). Application of an unbiased network and community clustering analysis on the undirected graph representing the relationship between each gene and which brain regions they were expressed in revealed three major communities. Based on their composition, these communities roughly mapped to the limbic system, cerebellum and cortex.

Though limbic system, cerebellar and cortical structures have previously been reported as exhibiting changes in autistic individuals compared to neurotypical controls (Mapelli et al. 2022; Ong and Fan 2023), the results presented here provide some novel insights. The first is that the regions identified here possibly hold special relevance to ASD from a mechanistically causal perspective. To date, many different brain regions have been reported to exhibit structural or functional differences in ASD including not only those reported here but also those within the frontal lobe, basal ganglia, thalamus and brainstem (Ha et al. 2015; Ayub et al. 2021; Seif et al. 2021). Given that ASD is largely genetic in origin (heritability of ~80% (Bai et al. 2019)), there are two possible hypotheses to explain the widespread changes in brain structure and function in autistic individuals: (1) that ASD‐associated genes are expressed in a homogenous distribution across the brain and thus influence the structure and function of all brain regions directly (primary effect) or (2) that ASD‐associated genes are enriched within specific brain regions, which are primarily impacted by polymorphisms and then consequentially influence the structure and function of other brain regions (secondary effect). The results presented here are highly supportive of the latter, though this should be interpreted with the recognition that ASD is heterogeneous both at the clinical and neurobiological level (Bitsika et al. 2018; Guo et al. 2022). It must be noted that clinical diversity of ASD likely arises from diverse patterns of genetic disruption affecting multiple neural systems. The finding that the ASD‐associated genes explored in this study are distributed across 11 discrete brain regions, rather than being confined to a single locus, suggests that different genetic variants may exert their primary effects in different regional circuits. This regional genetic architecture may also contribute to the observed subtypes of ASD, where distinct symptom clusters map onto dysfunction in specific neural systems. Thus, the multi‐regional enrichment pattern reported here is consistent with, and may mechanistically underlie, the phenotypical heterogeneity that is a hallmark of ASD.

The second major insight of this study is the identification of highly discrete targets for further study, some of which have yet to be empirically explored in individuals with ASD. Of the brain regions reported here, those that have already been identified as exhibiting structural or functional changes in autistic individuals include the CC (Bauman and Kemper 1985), Ve (Courchesne et al. 1994), FNL (Wegiel et al. 2010), CA1‐CA3 (Li et al. 2023), PCG (Ye et al. 2025) and OC (Jung et al. 2019). The aOFG and T‐O have not specifically been investigated, though changes to nearby or encompassing structures such as the orbitofrontal cortex and the temporo‐parietal‐occipital junction have been reported (Girgis et al. 2007; Bravo Balsa et al. 2024). The PG has yet to be investigated in autistic individuals, though one recent study employing a murine ASD model knocking down SCN2A has implicated a causal role of this brain region in learning and memory deficits (Keith et al. 2025). As such, the present study has identified several understudied brain regional targets for future investigations into ASD, including the aOFG, T‐O and PG.

### 4.2 ASD Syndromic Genes Specifically Enrich in the Orbitofrontal Region

One key insight provided by this study arose from the investigation of genes associated with syndromic versus non‐syndromic ASD. Of the 11 brain regions identified here, only one (the aOFG) displayed a statistically significant enrichment of syndromic genes. Finding equal representation of syndromic and non‐syndromic genes across most brain regions is an interesting observation because it arguably serves to reinforce the connection between ASD‐related syndromes and non‐syndromic ASD. It predicts that there is similar brain regional involvement for both syndromic and non‐syndromic ASD, despite genetically diverse aetiologies and variation in clinical presentation (Rylaarsdam and Guemez‐Gamboa 2019; Bitsika et al. 2018). The results presented here thus support the idea of there being a ‘module’ of brain regions (or regional microcircuits) underlying the development of ASD. Interestingly, this concept has been suggested previously at the genetic level based on results using murine models (Baudouin et al. 2012; Baudouin 2014). The findings reported here provide additional support to such a concept, extending observations to data from humans.

In stark contrast to most of the brain regions identified in this study is the aOFG, which was found to be particularly enriched for syndromic ASD‐associated genes. The aOFG is a subregion of the lateral orbitofrontal cortex, which has been shown to respond to compound cues and plays an active role in predicting outcomes given available multidimensional or multimodal information (Tegelbeckers et al. 2023). It is particularly important for determining how abstract rules are represented in the context of decision making (Tan et al. 2025), specifically with respect to adaptive regulation of social and emotional behaviour. Lesions to the orbitofrontal cortex have been reported to result in impairments in identifying the emotional content of vocalisations as well as the regulation of social behaviour (Hornak et al. 2003). The enrichment of genes associated with syndromic ASD specifically in the aOFG is of interest when considering that syndromic ASD often presents with more predictable (or ‘typical’) ASD‐related symptoms (Genovese and Butler 2024), as well as enhanced risk for co‐morbid neuropsychiatric disorders (Shaffer et al. 2023; Genovese and Ellerbeck 2022). Given the association of the aOFG with key symptomatic features of ASD (regulation and interpretation of multimodal emotional and social cues), this implies that the aOFG could be a locus of core ASD‐associated neuropsychiatric impairment. Additional work will be needed to validate this idea, with a specific focus on the aOFG region of the orbitofrontal cortex.

### 4.3 High‐Priority Genes Identify Key Signalling Pathways in ASD

Network analysis identified eight genes that may tentatively be considered as high priority for future study due to their broad relevance across multiple identified brain regions. Of these, five are difficult to interpret as their functions likely relate to DNA repair (*CHAMP1* (Li et al. 2025)), RNA decay (*UPF3B* (Lykke‐Andersen et al. 2000)), histone demethylation (*KDM5B* (Xiang et al. 2007)) and transcriptional regulation (*AFF2* (Melko et al. 2011) and *RFX3* (Sugiaman‐Trapman et al. 2018)), all of which are functionally dependent on the sequences they are associated with regulating. Of the remaining three, two major themes emerge: responsiveness to corticosteroids (*NR3C2* (Alvarez de la Rosa et al. 2024)) and regulation of synapses (*GABRB2* (Barki and Xue 2022) and *NBEA* (Lützenkirchen et al. 2024)). Because synaptic dysregulation in ASD has been described extensively (Lee et al. 2017), the implications of *NR3C2* will be specifically focused on here. It should bear mentioning, however, that *GABRB2* and *NBEA* are closely related to inhibitory synaptic transmission (Barki and Xue 2022; Lützenkirchen et al. 2024), which is congruent with previous reports indicating evolutionarily conserved relationships between ASD‐associated genes and the neurobiology of inhibitory neurons (Wang et al. 2018; Odierna, Stednitz, et al. 2025).

*NR3C2* was consistently one of the highest scored genes in the network across all three measures of nodal strength, betweenness centrality and closeness centrality, indicating potentially broad relevance across all identified brain regions. *NR3C2* encodes the mineralocorticoid receptor (MR), which plays key physiological roles in mammals relating to fluid balance and salt homeostasis via activation from the mineralocorticoid aldosterone (SPÄT and HUNYADY 2004). In the brain, MR is also intimately involved in the stress response as part of the hypothalamic–pituitary–adrenal axis (Paul et al. 2022). Because MR has a significantly higher affinity for cortisol than the glucocorticoid receptor, its involvement in the stress response is most important during times of baseline, or ‘low stress’, conditions. As such, it has been hypothesised that MR controls preparedness to stress and appraisal of stressors, rather than the reaction to the presence of stressors per se (Paul et al. 2022). This is particularly interesting to consider in the context of ASD given ongoing investigations into the role of anxiety in the symptomatology of autistic individuals. Anxiety is strongly co‐morbid with ASD (Bitsika et al. 2016), which extends to direct family members of affected individuals, such as parents of autistic children (Bitsika and Sharpley 2004). Though recent work has suggested that ASD symptoms are distinct from those relating specifically to ASD (Bitsika et al. 2025), the identification of *NR3C2* in this study implies a mechanistic and potentially causal relationship between neural mechanisms of stress hypervigilance and ASD, or at least some specific aspects of its symptomatology. One major recommendation that arises from this observation is that future studies should consider how resting levels of stressor factors into anxiety in ASD appraisal (with respect to core impairments in social comprehension), with less focus on cortisol itself, because changes at the level of the receptor and its signalling pathway are more likely to be relevant.

### 4.4 Implications of Findings Relating to Region‐Specific Sources of Gene Ontological Signatures

GO analysis is a powerful tool that has produced useful predictions regarding processes underlying expression of ASD phenotypes (Wang et al. 2018; Anney et al. 2011). One major limitation to how GO is applied in ASD, and in studies of the nervous system more broadly, is that samples for transcriptional analysis are often taken from singular discrete regions of the brain. An assumption is often made, or implied, that results gained from such approaches can be interpreted as being broadly representative. However, gene expression throughout the human brain is non‐uniform and can vary significantly between regions (Hawrylycz et al. 2012). This transcriptional diversity needs to be recognised to properly interpret how gene‐specific information, sourced from genomic technology, maps onto neurophysiology. Advances in single‐cell transcriptomics have been tackling this issue from a typology perspective, ascribing specific subsets of molecular processes to individual cell types (Wamsley et al. 2024). Some work has begun addressing regional differences (Gogate et al. 2024), though it has been much less explored.

Here, GO was applied to subsets of ASD‐associated genes on a by‐community basis, where each community was predominantly associated with groups of closely associated brain regions. Community 1 was comprised of the CA1, CA3, pCC and PG (limbic system); Community 2 was comprised of the CC, FNL and Ve (cerebellum); and Community 3 was comprised of the aOFG, PCG, T‐O and OC (cortex). The results indicated that each community was associated with different gene ontological terms, suggesting that each subset of brain regions may be responsible for largely distinct phenomena underlying the symptoms of ASD. Synaptic dysregulation, which has been frequently associated with ASD in the past, was represented most strongly in the limbic system. Interestingly, the GO term ‘vocalisation behaviour’ was significantly enriched in the Community 1 gene list, suggesting that the atypical vocalisations that are often observed as a symptom of ASD (Schoen et al. 2011) may have some origins in synaptically dysregulated limbic system neurons. The Community 2 gene list was instead enriched for terms describing histone methylation, particularly via the Sin3 and MLL1 complexes. These are tightly involved in development via cell cycle regulation and cellular differentiation (Guenther et al. 2005; Grzenda et al. 2009), suggesting that the cerebellum may be specifically vulnerable to neurodevelopmental threat compared to other regions of the brain. Lastly, the Community 3 cortical regions were enriched for terms specifically relating to axonal ionic currents via terms such as ‘voltage‐gated ion channel activity’, ‘node of Ranvier’ and ‘axon initial segment’. This result is interesting to consider in the context of models that describe ASD as a condition of atypical system‐level information processing (Bernhardt et al. 2025). Altered transmission dynamics of axonal action potentials would serve as a strong mechanistic driver of altered system‐wide information processing and specific enrichment of this in the cortex may underlie cognition and learning (Al‐Mazidi 2023). Whether this is true in ASD remains to be seen. As such, the results reported here support the need for additional work focusing on distinct dysfunctions in specific sub‐regions of the brain and their relevance to particular symptoms of ASD.

An important consideration when interpreting these findings is that gene expression patterns vary substantially across the lifespan (Kumar et al. 2013). The transcriptomic data used in this study were derived from older adult post‐mortem brains. The regional enrichment patterns identified here may not be reflective of developmental contexts. This is particularly relevant for ASD, which is a neurodevelopmental condition whose atypicalities can be observed prenatally (Regev et al. 2022). Dynamic changes in gene expression during critical developmental windows may result in different regional vulnerability profiles at different life stages. Future studies employing developmental transcriptomic datasets could reveal whether the brain regional enrichments identified in this study are stable across the lifespan or whether distinct regions become vulnerable at specific developmental timepoints. Such temporal resolution would be invaluable for identifying critical periods for early intervention and could inform age‐specific biomarker strategies.

### 4.5 Broader Implications for Neurodevelopmental Disorders

Although this study focused specifically on ASD, it is important to recognise that many of the SFARI‐listed ASD‐associated genes are also implicated in other neurodevelopmental disorders, particularly ID and epilepsy (Pattie and Iffland 2025). This genetic overlap suggests shared neurobiological mechanisms across neurodevelopmental conditions and raises the possibility that the brain regional enrichments identified here may have relevance beyond ASD alone. For example, genes associated with epilepsy often involve ion channel dysfunction and synaptic excitability, which are processes that were enriched in the cortical regions identified in this study (Pattie and Iffland 2025). Similarly, many syndromic forms of ID share genetic aetiology with syndromic ASD (Fernandez and Scherer 2017), and the enrichment of syndromic genes in the aOFG may reflect a common substrate for cognitive and social impairments observed across multiple neurodevelopmental diagnoses. Future comparative studies applying ATLANTE to gene lists from other neurodevelopmental disorders could reveal whether distinct conditions converge on similar brain regional vulnerabilities or whether each disorder exhibits a unique regional signature. Such work would refine our understanding of transdiagnostic mechanisms and could inform the development of interventions applicable across multiple neurodevelopmental conditions.

## 5 Conclusion

This study employed ATLANTE, a validated gene–brain region analysis pipeline, to identify 11 discrete brain regions significantly enriched for high expression of ASD‐associated genes. Network and community clustering analyses revealed that ASD‐associated genes segregate into three major communities corresponding to distinct neurobiological processes: synaptic dysfunction in limbic regions, histone modification in cerebellar regions and axonal ion channel regulation in cortical regions. Network node analysis identified eight high‐priority genes that exhibit broad relevance across multiple brain regions and may represent particularly promising targets for biomarker development and therapeutic intervention. The findings of this study challenge the assumption that signatures derived from single‐region analyses are broadly representative, highlighting the need for region‐specific investigations. Moreover, they provide a refined neuroanatomical framework for understanding ASD pathophysiology and offer discrete, empirically supported targets for future diagnostic and therapeutic research.

## Author Contributions

**G. Lorenzo Odierna:** conceptualization, formal analysis, methodology, validation, visualization, writing – original draft, writing – review and editing. **Vicki Bitsika:** conceptualization, investigation, project administration, resources, validation, writing – review and editing. **Christopher F. Sharpley:** data curation, investigation, validation, writing – review and editing.

## Funding

The authors have nothing to report.

## Ethics Statement

No animal or human participants were involved in this research.

## Consent

No human patients were involved in this research.

## Conflicts of Interest

The authors disclose no conflicts of interest.

## Supporting information


**Table S1:** ASD‐associated genes used in this study.

## Data Availability

Data used in this study were derived from the following resources available in the public domain: Human Proteome Atlas, www.proteinatlas.org; SFARI GENE, https://gene.sfari.org/.
