# Supplementary material for: Autism Spectrum Disorder–Associated Genes Enrich in Discrete Cortical, Limbic and Cerebellar Brain Regions
Source: Eur J Neurosci. 2026 Jul 24;64(2):e70638. doi: 10.1111/ejn.70638 (PMC13400753; doi:10.1111/ejn.70638)
Supplement: Supplementary file 1 — Table S1: ASD‐associated genes used in this study. [file EJN-64-0-s001.docx]

**Supplemental information**

| Table S1. ASD-associated genes used in this study | | | |
| --- | --- | --- | --- |
| **Enseml ID** | **Gene symbol** | **Syndromic** | **Enriched** |
| ENSG00000164163 | ABCE1 | NO | NO |
| ENSG00000075624 | ACTB | YES | NO |
| ENSG00000101126 | ADNP | YES | YES |
| ENSG00000239900 | ADSL | YES | YES |
| ENSG00000155966 | AFF2 | NO | YES |
| ENSG00000126705 | AHDC1 | YES | YES |
| ENSG00000112294 | ALDH5A1 | YES | YES |
| ENSG00000145362 | ANK2 | NO | YES |
| ENSG00000151150 | ANK3 | NO | YES |
| ENSG00000167522 | ANKRD11 | YES | YES |
| ENSG00000140350 | ANP32A | NO | YES |
| ENSG00000042753 | AP2S1 | NO | YES |
| ENSG00000134287 | ARF3 | NO | YES |
| ENSG00000131089 | ARHGEF9 | YES | YES |
| ENSG00000049618 | ARID1B | YES | YES |
| ENSG00000004848 | ARX | YES | YES |
| ENSG00000116539 | ASH1L | NO | YES |
| ENSG00000141431 | ASXL3 | YES | YES |
| ENSG00000085224 | ATRX | NO | YES |
| ENSG00000158321 | AUTS2 | NO | NO |
| ENSG00000123636 | BAZ2B | NO | YES |
| ENSG00000103507 | BCKDK | NO | NO |
| ENSG00000119866 | BCL11A | YES | YES |
| ENSG00000157764 | BRAF | YES | YES |
| ENSG00000174672 | BRSK2 | YES | YES |
| ENSG00000141837 | CACNA1A | YES | YES |
| ENSG00000151067 | CACNA1C | YES | YES |
| ENSG00000198216 | CACNA1E | NO | YES |
| ENSG00000157445 | CACNA2D3 | NO | YES |
| ENSG00000108509 | CAMTA2 | NO | YES |
| ENSG00000135387 | CAPRIN1 | NO | NO |
| ENSG00000147044 | CASK | NO | NO |
| ENSG00000130940 | CASZ1 | NO | NO |
| ENSG00000008086 | CDKL5 | YES | YES |
| ENSG00000101489 | CELF4 | NO | YES |
| ENSG00000198824 | CHAMP1 | YES | YES |
| ENSG00000173575 | CHD2 | YES | YES |
| ENSG00000170004 | CHD3 | YES | YES |
| ENSG00000171316 | CHD7 | YES | YES |
| ENSG00000100888 | CHD8 | YES | YES |
| ENSG00000079432 | CIC | NO | NO |
| ENSG00000088038 | CNOT3 | YES | YES |
| ENSG00000102879 | CORO1A | NO | YES |
| ENSG00000149532 | CPSF7 | NO | YES |
| ENSG00000005339 | CREBBP | YES | YES |
| ENSG00000009307 | CSDE1 | YES | NO |
| ENSG00000101266 | CSNK2A1 | YES | YES |
| ENSG00000102974 | CTCF | YES | YES |
| ENSG00000168036 | CTNNB1 | NO | YES |
| ENSG00000036257 | CUL3 | NO | YES |
| ENSG00000215301 | DDX3X | YES | NO |
| ENSG00000177030 | DEAF1 | YES | YES |
| ENSG00000172893 | DHCR7 | YES | NO |
| ENSG00000160305 | DIP2A | NO | NO |
| ENSG00000132535 | DLG4 | NO | YES |
| ENSG00000104936 | DMPK | YES | NO |
| ENSG00000119772 | DNMT3A | YES | YES |
| ENSG00000092964 | DPYSL2 | NO | NO |
| ENSG00000171587 | DSCAM | NO | NO |
| ENSG00000197102 | DYNC1H1 | NO | YES |
| ENSG00000157540 | DYRK1A | YES | YES |
| ENSG00000108001 | EBF3 | YES | NO |
| ENSG00000181090 | EHMT1 | YES | YES |
| ENSG00000130811 | EIF3G | NO | NO |
| ENSG00000196361 | ELAVL3 | NO | YES |
| ENSG00000100393 | EP300 | YES | YES |
| ENSG00000102081 | FMR1 | YES | YES |
| ENSG00000176165 | FOXG1 | YES | NO |
| ENSG00000114861 | FOXP1 | YES | YES |
| ENSG00000128573 | FOXP2 | NO | YES |
| ENSG00000145864 | GABRB2 | NO | YES |
| ENSG00000166206 | GABRB3 | NO | YES |
| ENSG00000131095 | GFAP | NO | NO |
| ENSG00000146830 | GIGYF1 | NO | YES |
| ENSG00000204120 | GIGYF2 | NO | YES |
| ENSG00000127955 | GNAI1 | YES | NO |
| ENSG00000120251 | GRIA2 | NO | YES |
| ENSG00000176884 | GRIN1 | NO | YES |
| ENSG00000183454 | GRIN2A | NO | YES |
| ENSG00000273079 | GRIN2B | NO | YES |
| ENSG00000115677 | HDLBP | NO | NO |
| ENSG00000173064 | HECTD4 | NO | YES |
| ENSG00000010818 | HIVEP2 | YES | YES |
| ENSG00000126945 | HNRNPH2 | NO | YES |
| ENSG00000153187 | HNRNPU | YES | YES |
| ENSG00000174775 | HRAS | NO | NO |
| ENSG00000124313 | IQSEC2 | YES | YES |
| ENSG00000119669 | IRF2BPL | YES | NO |
| ENSG00000120071 | KANSL1 | YES | YES |
| ENSG00000167216 | KATNAL2 | NO | NO |
| ENSG00000158445 | KCNB1 | YES | YES |
| ENSG00000184156 | KCNQ3 | NO | YES |
| ENSG00000089094 | KDM2B | NO | YES |
| ENSG00000120733 | KDM3B | YES | NO |
| ENSG00000117139 | KDM5B | NO | YES |
| ENSG00000126012 | KDM5C | NO | YES |
| ENSG00000132510 | KDM6B | NO | YES |
| ENSG00000170871 | KIAA0232 | NO | NO |
| ENSG00000076321 | KLHL20 | NO | YES |
| ENSG00000118058 | KMT2A | YES | YES |
| ENSG00000055609 | KMT2C | YES | NO |
| ENSG00000005483 | KMT2E | YES | YES |
| ENSG00000110066 | KMT5B | NO | YES |
| ENSG00000198728 | LDB1 | NO | YES |
| ENSG00000148948 | LRRC4C | NO | YES |
| ENSG00000099949 | LZTR1 | NO | NO |
| ENSG00000165509 | MAGEC3 | NO | NO |
| ENSG00000254585 | MAGEL2 | YES | NO |
| ENSG00000166963 | MAP1A | NO | YES |
| ENSG00000204406 | MBD5 | YES | YES |
| ENSG00000125505 | MBOAT7 | YES | NO |
| ENSG00000169057 | MECP2 | YES | YES |
| ENSG00000108510 | MED13 | YES | YES |
| ENSG00000123066 | MED13L | YES | YES |
| ENSG00000081189 | MEF2C | YES | YES |
| ENSG00000134138 | MEIS2 | YES | NO |
| ENSG00000150051 | MKX | NO | YES |
| ENSG00000005302 | MSL3 | YES | YES |
| ENSG00000198793 | MTOR | YES | NO |
| ENSG00000005810 | MYCBP2 | NO | YES |
| ENSG00000186487 | MYT1L | NO | YES |
| ENSG00000164134 | NAA15 | YES | YES |
| ENSG00000160877 | NACC1 | YES | YES |
| ENSG00000172915 | NBEA | YES | YES |
| ENSG00000061676 | NCKAP1 | NO | YES |
| ENSG00000084676 | NCOA1 | NO | YES |
| ENSG00000050030 | NEXMIF | NO | NO |
| ENSG00000196712 | NF1 | YES | YES |
| ENSG00000164190 | NIPBL | YES | YES |
| ENSG00000169992 | NLGN2 | NO | NO |
| ENSG00000196338 | NLGN3 | NO | YES |
| ENSG00000146938 | NLGN4X | NO | YES |
| ENSG00000151623 | NR3C2 | YES | YES |
| ENSG00000153234 | NR4A2 | NO | YES |
| ENSG00000179915 | NRXN1 | NO | YES |
| ENSG00000110076 | NRXN2 | NO | YES |
| ENSG00000021645 | NRXN3 | NO | YES |
| ENSG00000165671 | NSD1 | YES | YES |
| ENSG00000113569 | NUP155 | NO | YES |
| ENSG00000175115 | PACS1 | YES | YES |
| ENSG00000171759 | PAH | NO | NO |
| ENSG00000196092 | PAX5 | NO | NO |
| ENSG00000114054 | PCCB | YES | NO |
| ENSG00000165194 | PCDH19 | YES | YES |
| ENSG00000109118 | PHF12 | NO | YES |
| ENSG00000197724 | PHF2 | NO | YES |
| ENSG00000135365 | PHF21A | YES | YES |
| ENSG00000118482 | PHF3 | NO | YES |
| ENSG00000146247 | PHIP | YES | YES |
| ENSG00000143442 | POGZ | YES | YES |
| ENSG00000085998 | POMGNT1 | YES | NO |
| ENSG00000108819 | PPP1R9B | NO | YES |
| ENSG00000112640 | PPP2R5D | YES | YES |
| ENSG00000011485 | PPP5C | NO | YES |
| ENSG00000126464 | PRR12 | YES | YES |
| ENSG00000183530 | PRR14L | NO | YES |
| ENSG00000108671 | PSMD11 | NO | YES |
| ENSG00000197170 | PSMD12 | YES | YES |
| ENSG00000163636 | PSMD6 | NO | YES |
| ENSG00000165186 | PTCHD1 | NO | YES |
| ENSG00000171862 | PTEN | YES | YES |
| ENSG00000112655 | PTK7 | NO | NO |
| ENSG00000179295 | PTPN11 | YES | NO |
| ENSG00000108557 | RAI1 | YES | YES |
| ENSG00000258436 | RNASE12 | NO | YES |
| ENSG00000170471 | RALGAPB | NO | YES |
| ENSG00000189056 | RELN | YES | YES |
| ENSG00000142599 | RERE | NO | YES |
| ENSG00000080298 | RFX3 | NO | YES |
| ENSG00000079841 | RIMS1 | YES | NO |
| ENSG00000202538 | RNU4-2 | YES | YES |
| ENSG00000198963 | RORB | NO | YES |
| ENSG00000079102 | RUNX1T1 | YES | YES |
| ENSG00000182568 | SATB1 | YES | YES |
| ENSG00000144285 | SCN1A | NO | YES |
| ENSG00000136531 | SCN2A | NO | YES |
| ENSG00000196876 | SCN8A | NO | YES |
| ENSG00000152217 | SETBP1 | YES | YES |
| ENSG00000099381 | SETD1A | NO | YES |
| ENSG00000181555 | SETD2 | YES | YES |
| ENSG00000168137 | SETD5 | NO | NO |
| ENSG00000162105 | SHANK2 | YES | YES |
| ENSG00000251322 | SHANK3 | YES | YES |
| ENSG00000169375 | SIN3A | NO | YES |
| ENSG00000157933 | SKI | YES | YES |
| ENSG00000157103 | SLC6A1 | YES | YES |
| ENSG00000198689 | SLC9A6 | YES | YES |
| ENSG00000080503 | SMARCA2 | NO | YES |
| ENSG00000127616 | SMARCA4 | YES | YES |
| ENSG00000139613 | SMARCC2 | YES | YES |
| ENSG00000159140 | SON | NO | NO |
| ENSG00000100485 | SOS2 | YES | NO |
| ENSG00000134532 | SOX5 | NO | NO |
| ENSG00000021574 | SPAST | NO | YES |
| ENSG00000080603 | SRCAP | NO | YES |
| ENSG00000182934 | SRPRA | YES | YES |
| ENSG00000136854 | STXBP1 | NO | YES |
| ENSG00000008056 | SYN1 | YES | YES |
| ENSG00000197283 | SYNGAP1 | YES | YES |
| ENSG00000170921 | TANC2 | YES | NO |
| ENSG00000160551 | TAOK1 | NO | YES |
| ENSG00000154114 | TBCEL | YES | YES |
| ENSG00000145348 | TBCK | NO | YES |
| ENSG00000177565 | TBL1XR1 | NO | YES |
| ENSG00000136535 | TBR1 | YES | YES |
| ENSG00000100207 | TCF20 | YES | YES |
| ENSG00000196628 | TCF4 | NO | NO |
| ENSG00000148737 | TCF7L2 | NO | YES |
| ENSG00000120156 | TEK | NO | NO |
| ENSG00000140332 | TLE3 | YES | YES |
| ENSG00000146872 | TLK2 | NO | NO |
| ENSG00000101337 | TM9SF4 | YES | YES |
| ENSG00000131653 | TRAF7 | NO | YES |
| ENSG00000113595 | TRIM23 | NO | YES |
| ENSG00000038382 | TRIO | YES | YES |
| ENSG00000153827 | TRIP12 | YES | YES |
| ENSG00000165699 | TSC1 | YES | NO |
| ENSG00000103197 | TSC2 | NO | YES |
| ENSG00000179981 | TSHZ1 | NO | YES |
| ENSG00000121297 | TSHZ3 | NO | YES |
| ENSG00000143569 | UBAP2L | YES | YES |
| ENSG00000114062 | UBE3A | NO | NO |
| ENSG00000159459 | UBR1 | YES | YES |
| ENSG00000125351 | UPF3B | YES | YES |
| ENSG00000124486 | USP9X | NO | NO |
| ENSG00000136451 | VEZF1 | YES | NO |
| ENSG00000132549 | VPS13B | YES | YES |
| ENSG00000095787 | WAC | NO | NO |
| ENSG00000163625 | WDFY3 | YES | YES |
| ENSG00000100811 | YY1 | YES | YES |
| ENSG00000181722 | ZBTB20 | NO | NO |
| ENSG00000173276 | ZBTB21 | YES | YES |
| ENSG00000101040 | ZMYND8 | YES | YES |
| ENSG00000188994 | ZNF292 | YES | NO |
| ENSG00000148143 | ZNF462 | NO | NO |
